# Antioxidant and Antitumor Activities of Novel Quercetin-Loaded Electrospun Cellulose Acetate/Polyethylene Glycol Fibrous Materials

**DOI:** 10.3390/antiox9030232

**Published:** 2020-03-11

**Authors:** Nikoleta Stoyanova, Mariya Spasova, Nevena Manolova, Iliya Rashkov, Ani Georgieva, Reneta Toshkova

**Affiliations:** 1 Laboratory of Bioactive Polymers, Institute of Polymers, Bulgarian Academy of Sciences, Acad. G. Bonchev St, bl. 103A, BG-1113 Sofia, Bulgaria; nstoyanova@polymer.bas.bg (N.S.); manolova@polymer.bas.bg (N.M.); 2 Institute of Experimental Morphology, Pathology and Anthropology with Museum, Bulgarian Academy of Sciences, Acad. G. Bonchev St, bl. 25, BG-1113 Sofia, Bulgaria; ageorgieva@bas.bg (A.G.); rtoshkova@bas.bg (R.T.)

**Keywords:** quercetin, electrospinning, cellulose acetate, polyethylene glycol, antioxidant activity, radical scavenging, antitumor effect, HeLa, SH-4

## Abstract

The aim of present study was to obtain novel fibrous materials based on cellulose derivative and polyethylene glycol loaded with natural biologically active compound quercetin by electrospinning. Several methods including scanning electron microscopy (SEM), IR spectroscopy, X-ray diffraction analysis (XRD), water contact angle measurements, differential scanning calorimetry (DSC), and UV-VIS spectroscopy were utilized to characterize the obtained materials. The incorporation of polyethylene glycol in the fibrous material resulted in increased hydrophilicity and burst release of quercetin from the fibers. Quercetin-containing fibrous mats exhibited high antioxidant activity as estimated by DPPH free radical scavenging method. In vitro tests with HeLa tumor cells and SH-4 melanoma skin cells were performed in order to determine the cytotoxicity of the novel materials. It was found that the fibrous CA/PEG/QUE materials exhibited high cytotoxic effect against both cell lines. Therefore, the novel polymeric materials containing quercetin are promising candidates for biomedical and pharmaceutical applications.

## 1. Introduction

Plants are a valuable source of bioactive compounds such as terpenes, phenolic compounds, essential oils, alkaloids, etc. Some plant extracts possess antidiabetic, antihyperlipidemic, antioxidant, and anti-inflammatory activities [1]. Quercetin occurs abundantly in a variety of fruits and vegetables [2]. This biologically active compound exhibits remarkable antioxidant [3,4,5], anti-inflammatory [6], antibacterial [7,8], and anti-tumor [9] activities, and it possesses high anti-allergic activity [10]. However, quercetin is almost insoluble in water, sparingly soluble in gastrointestinal fluids, and it is metabolized by human intestinal bacteria [11,12]. For that reason, it is necessary to develop suitable carriers of quercetin capable to enhance its solubility in water, which will lead to enhanced bioavailability and thus to improved biological activity. An approach to circumvent the low water solubility of quercetin is its incorporation into amorphous polymer matrices.

Various carriers have been investigated as potential carriers for plant-derived products. Madaan et al. have used polyamidoamine (PAMAM) dendrimers as oral drug delivery carriers for quercetin and have shown improved aqueous solubility of the incorporated flavonoid [13]. In order to enhance stability and solubility of quercetin, liposome-chitosan hydrogel beads have been developed by the injection gelation method [14]. The fabricated spheres have three-dimensional network structure with improved chemical stability of the bioactive compound. Multiphase hydrogel system incorporated with quercetin loaded liposomes for wound healing applications has been fabricated as well. The prepared hydrogel combines the effect to control the release of quercetin with haemocompatibility and good mechanical properties as well as accelerates wound healing [15].

In recent years, electrospinning has proven to be a promising technique for the fabrication of polymeric drug delivery carriers [16]. This is due to the fact that when the diameters of the polymer fibers decrease to micrometers or nanometers, some interesting properties of the materials occur. These include high-surface-area-to-volume ratio, flexible surface modification and modulation of the drug release profile, improved therapeutic effect and mechanical properties, and reduced undesirable side effects. Moreover, electrospinning offers high loading capacity and high encapsulation efficiency of the incorporated drugs.

Electrospun materials are suitable carriers of biologically active compounds of both natural [17] and synthetic origin [18]. Quercetin has been incorporated in polymer fibers from polycaprolactone, poly(vinyl pyrrolidone) [19], polylactic acid, zein [20], polylactic-*co*-glycolic acid [21], and ethyl cellulose [22].

The aim of this work was to fabricate and characterize electrospun materials of cellulose acetate (CA) and polyethylene glycol (PEG) for quercetin delivery. The composition of the polymer matrix was selected so as to improve the release of quercetin from the fibers. The antioxidant activity of the obtained novel materials as well as the behavior of the materials in contact with HeLa tumor cells and SH-4 melanoma skin cells were investigated.

## 2. Materials and Methods

### 2.1. Materials

Cellulose acetate (CA) with ${\bar{M}}_{n}$ = 30,000 g/mol and DS 39.8% was purchased from Aldrich (St. Louis, MO, USA). Polyethylene glycol (PEG) with (Mr = 1900–2200 g/mol) was obtained from Fluka (Buchs, Switzerland). Quercetin (QUE ≥95%; Sigma–Aldrich, St. Louis, MO, USA) and Tween 80 (Acros Organics, Amsterdam, Netherlands) were used. Acetone (Sigma–Aldrich, Darmstadt, Germany) and ethanol (Sigma–Aldrich, ≥99.8% (GS)) of analytical grade of purity were used. 2,2-Diphenyl-1-picrylhydrazyl (DPPH) from Sigma–Aldrich (Darmstadt, Germany), 3-(4.5-dimethylthiazol-2-yl)-2.5-diphenyltetrazolium bromide (MTT; Sigma–Aldrich, Darmstadt, Germany), ethidium bromide (EtBr; Sigma Chemical, Balcatta, Australia), acridine orange (AO; Sigma Chemical, Balcatta, Australia) and 4′,6-diamidino-2-phenylindole dihydrochloride (DAPI, Sigma–Aldrich, Darmstadt, Germany) were of analytical grade of purity and were used without further purification. All culture reagents Dulbecco’s Modified Eagle’s Medium (DMEM) (Sigma–Aldrich, Schnelldorf, Germany), fetal bovine serum (FBS) (Gibso/BRL, Grand Island, NY, USA), glutamine, penicillin, and streptomycin (LONZA, Cologne, Germany) were used as received. The disposable consumables were supplied by Orange Scientific, Braine-l’Alleud, Belgium. HeLa human cervical cancer cells (ATCC, CCL-2) and SH-4 melanoma cells (ATCC, CRL-7724) were obtained from the American Type Cultures Collection (ATCC, Rockville, MD, USA).

### 2.2. Preparation of Fibrous Materials by Electrospinning

Fibrous materials of different composition were obtained by electrospinning: CA, CA/PEG, and CA/PEG/QUE. Three types of solutions were prepared for electrospinning in acetone/water 80/20 *v/v*: (i) CA, (ii) CA/PEG (80/20 *w/w*), and (iii) CA/PEG (80/20 *w/w*) with QUE (10 wt% in respect to total polymer weight). The total polymer concentration was 10 wt%.

The electrospinning apparatus used for the preparation the fibers consisted of a pump with adjustable speed and 5 mL syringe (12 mm internal diameter) fitted to a metal needle with a tip (size: 20GX1½) connected to the positively charged electrode of a high voltage power supply (up to 30 kV). The spinning solutions were placed in the syringe. The electrospinning was performed at 21 °C, relative humidity −50%, applied voltage of 25 kV and a tip-to-collector distance of 15 cm to the grounded rotating metal cylindrical collector (1000 rpm). The constant delivery rate of the spinning solutions of 3 mL/h was provided by a pump Syringe Pump NE-300 (New Era Pump Systems, Inc., New York, NY, USA).

### 2.3. Characterization of the Fibrous Materials

The morphology of the fibrous materials was evaluated by scanning electron microscopy (SEM). SEM analyses were performed on a Jeol JSM-5510 scanning electron microscope (Tokyo, Japan). The samples were vacuum-coated with gold by cathode sputtering using a Jeol JFC-1200 apparatus.

Mean fiber diameter was estimated by Image J software [23] (U.S. National Institutes of Health, Bethesda, MD, USA) by measuring the diameters of at least 60 fibers. The criteria for overall evaluation of electrospun materials were used to evaluate their morphology [24].

The materials were analyzed by Fourier transform infrared spectroscopy (Shimadzu Co., Kyoto, Japan), supplied with a MIRacle ATR device (diamond crystal; depth of penetration of the IR beam into the sample: approximately 2 µm) (PIKE Technologies, Fitchburg, WI, USA) in a scanning range of 600–4000 cm^−1^ with a resolution of 4 cm^−1^. All spectra were corrected for H_2_O and CO_2_ using an IRsolution software programme.

The thermal characteristics of the materials were analyzed by differential scanning calorimetry (DSC). Samples were heated in the temperature range from 0 to 380 °C at a heating rate of 10 °C/min in nitrogen atmosphere (TA Instruments, DSC Q2000, New Castle, DE, USA).

Computer-controlled D8 Bruker Advance ECO powder diffractometer with filtered Cu Kα radiation was used to perform X-ray diffraction (XRD) analyses. Data were collected in the 2θ range from 5° to 60° with a step of 0.02° and counting time of 1 s step^−1^.

Easy Drop DSA20E KRÜSS GmbH apparatus (Hamburg, Germany) was used to determine the water contact angle values of fibrous materials. Deionized water (10 µL) was dropped on the surface of fibrous specimens. The water contact angle value was assessed after averaging at least 10 measurements for each specimen.

Quercetin content in the fibrous materials was determined by dissolving samples (1 cm^2^) in 10 mL of acetone/water (80/20 *v/v*). Then the absorbance at 373 nm was measured using a DU 800 spectrophotometer UV (Beckman Coulter, Brea, CA, USA). The QUE loading efficiency was calculated from the following equation:

Loading efficiency = (amount of loaded QUE/amount of QUE in the feed) × 100%

Quercetin release profile was studied in vitro at 37 °C in acetate buffer at pH 5.5, constant ionic strength 0.1 (CH_3_COONa/CH_3_COOH) containing Tween 80 (acetate buffer/Tween 80 = 99.2/0.8 *v/v*). The tested mats were immersed in 100 mL buffer solution stirred at 150 rpm with an electromagnetic stirrer. Aliquots of the test solution were withdrawn at determined time intervals and the amount of QUE in the release medium was determined from the absorbance at 373 nm by using DU 800 UV spectrophotometer (Beckman Coulter). A calibration curve absorbance/QUE concentration (correlation coefficient *R* = 0.999) was used for the calculations.

The antioxidant activity of the materials was determined using the 2,2-diphenyl-1-picrylhydrazyl (DPPH) radical scavenging assay. For this purpose, ethanol solution of the DPPH (2.5 mL) with a concentration of 1 × 10^−4^ M was added to 0.5 mL of ethanol solution of QUE (0.5 mg QUE). Fibrous mats of CA/PEG (5 mg mat) and CA/PEG/QUE (5 mg mat containing 0.5 mg QUE) were immersed in 3 mL of DPPH solution in ethanol. The as-prepared mixed solutions were kept in the dark at 20 °C for 30 min. The antioxidant activity was evaluated by measuring the absorbance of the solutions at 517 nm using a DU 800 UV-vis spectrophotometer (Beckman Coulter), to detect the amount of DPPH radicals remaining in the solution. The antioxidant activity (AA%) was calculated using the following equation:

$$\mathrm{Inhibition},\;\mathrm{AA},\%\;=\;\left[\frac{(A_{\mathrm{DPPH}}-A_{\mathrm{sample}})}{A_{\mathrm{DPPH}}}\right]\;\times \;100\;$$

where A_sample_-absorption at 517 nm for DPPH• solution after the addition of the solution containing QUE or fibrous materials, A_DPPH•_-absorption at 517 nm for DPPH• solution. All experiments were performed in triplicate.

### 2.4. MTT Cytotoxicity Assay

HeLa cells and SH-4 (Homo sapiens skin melanoma) (from ATCC, Rockville, MD, USA) were cultured in DMEM supplemented with 10% FBS, 100 U/mL penicillin, and 0.1 mg/mL streptomycin in a CO_2_ incubator at 37 °C and 5% CO_2_. Cell were trypsinized after reaching 80–90% confluence, by 0.25% Trypsin-EDTA and counted with a hemocytometer. Cells were placed in a 96-well plate with a concentration of 1 × 10^5^ cells per well. The medium was changed after overnight incubation at 37 °C in humidified air with 5% CO_2_ to facilitate cells attachment. HeLa and SH-4 cells were placed in contact with fibrous materials (CA/PEG and CA/PEG/QUE) for 24 and 48 h. HeLa cells and SH-4 incubated alone and in the presence of QUE were used as controls. MTT assay was used to determine the effect of different fibrous materials on cell viability [25]. Each variant was assayed by five measurements. After culturing in the presence of mats, the HeLa and SH-4 cells were washed twice with PBS (pH 7.4) and further incubated with 100 μL of MTT working solution (Sigma Chemical) at 37 °C for 3 h. The supernatants were aspirated, and 100 μL of lysing solution (DMSO/ethanol 1:1) was added to each well to dissolve the resulting formazan. MTT assay reading was performed using ELISA plate reader (TECAN, SunriseTM, Grödig/Salzburg, Austria). The percentage of cell viability was calculated as follows:

cell viability (%) = OD_570_ (experimental)/OD_570_ (control) × 100

### 2.5. Study of the Effect of the Fibrous Mats on HeLa and SH-4 Cells Using Fluorescence Microscopy

#### Double Staining Assay with AO–EtBr

HeLa and SH-4 cells were plated on glass lamellas (12 mm Ø) placed on the bottom of 24-well plates, at a concentration of 2 × 10^5^ cells mL^−1^, and incubated at 37 °C for 24 h in a CO_2_ incubator to form a monolayer. After that, CA/PEG and CA/PEG/QUE fibrous mats sterilized by UV-light were placed in 24-well plates for further 24 h incubation. Untreated tumor cells were used as negative control and QUE-treated HeLa or SH-4 cells were used as positive control. After 24 h of incubation, the mats were removed and glass lamellas were washed twice with phosphate-buffered saline (PBS, pH 7.4) to remove unattached cells, then stained with AO and EtBr (equal volume), mounted onto glass slides, and processed immediately for fluorescent microscopy (Leika DM 5000B, Wetzlar, Germany). Images were recorded by a digital camera connected to the microscope.

### 2.6. DAPI-Staining

The nuclear morphology of the HeLa or SH-4 cells was observed using 4′,6-diamidino-2-phenylindole (DAPI) staining. 1 × 10^5^ cells/well were seeded on glass cover sleeps in 24-well tissue culture plates, and were cultured in the presence of the fibrous mats for 24 h after overnight incubation in a CO_2_ incubator. Cells were fixed with 3% paraformaldehyde at room temperature and subsequently stained for cell nuclei observation by a fluorescence microscope (Leica DM 5000B, Wetzlar, Germany).

## 3. Results

In our previous study, it was found that cellulose acetate (CA) fibers, electrospun from a solution with a polymer concentration of 10 wt.%, were defect-free, homogeneous, and with an average fiber diameter of 780 nm. The obtained CA-based fibers have been shown to be suitable carriers of the natural polyphenolic compound curcumin [17]. We have also shown that the incorporation of water-soluble polymers into the cellulose acetate-based polymeric matrix enhances the solubility of curcumin, its wetting ability, and release [26].

**Figure 1:**
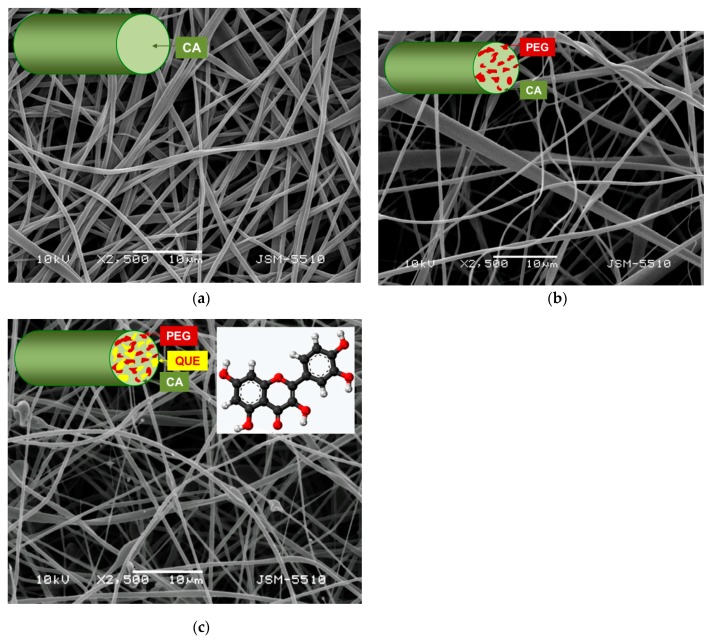
Micrographs of the fibers: (**a**) CA, (**b**) СА/PEG, and (**c**) СА/PEG/QUE and their schematic representation (insets).

The types of the fibers obtained in the present study by electrospinning of (*i*) CA solution, (*ii*) CA/PEG blend solution, and (*iii*) CA/PEG/QUE blend solution are schematically represented in Figure 1 (insets). The selected polymer carriers, solvent system, and process parameters enabled obtaining fibrous materials with quantitative loading efficiency (~100%).

### 3.1. Preparation of Fibrous Mats by Electrospinning

SEM micrographs of the obtained CA, CA/PEG, and CA/PEG/QUE fibrous materials are shown in Figure 1. Electrospinning of CA solution under the selected conditions resulted in obtaining defect-free fibers with mean fiber diameter of 780 ± 80 nm (Figure 1a). It was found that the incorporation of PEG resulted in decrease of the mean fiber diameter. The mean fiber diameter of CA/PEG fibrous material was 530 ± 150 nm (Figure 1b). This is probably due to the presence of PEG, which lowers the solution viscosity and leads to the fabrication of fibers with smaller diameters. Further decrease in the mean fiber diameters to 390 ± 150 nm was observed in the case of CA/PEG/QUE fibrous materials (Figure 1c).

### 3.2. IR Spectra of Fibrous Materials

**Figure 2:**
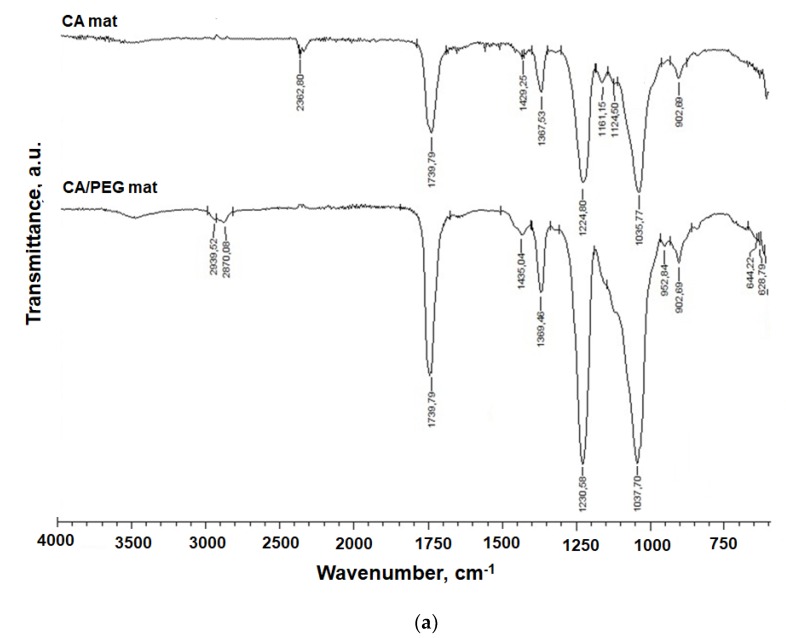

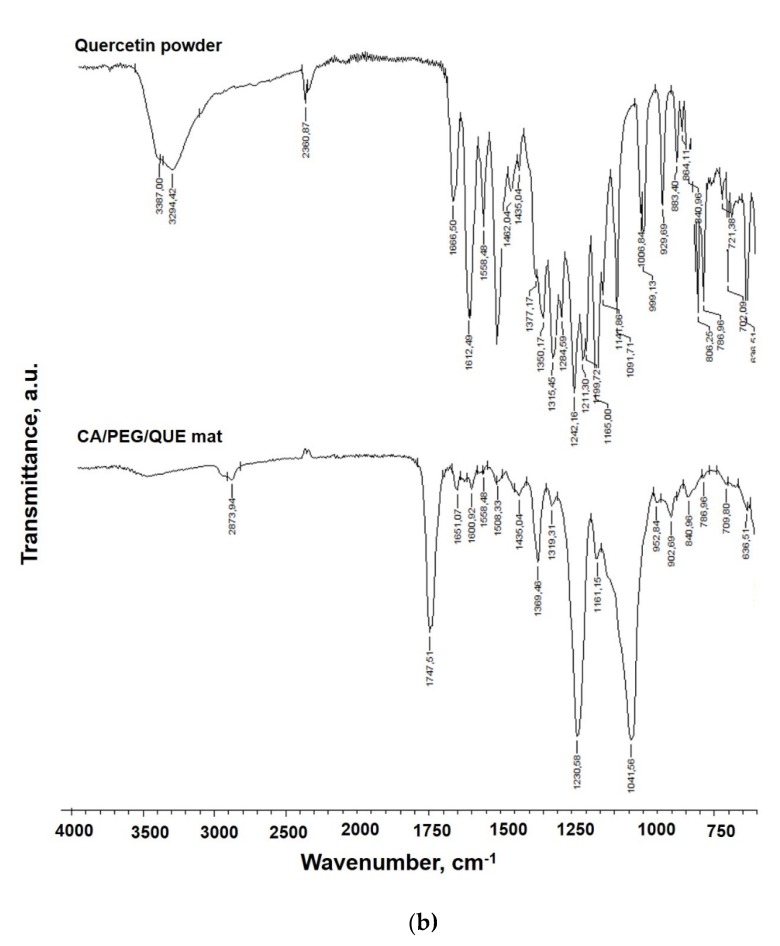
IR spectra of: (**a**) CA and CA/PEG and (**b**) CA/ PEG/QUE fibers and quercetin (powder).

The FTIR spectra of quercetin (powder), CA mats, CA/PEG mats, and CA/PEG/QUE mats are presented in Figure 2. Characteristic bands for C=O functional groups at 1740 cm^−1^, for CH_3_ groups at 1369 and 1226 cm^−1^, as well as ether C-O-C groups at 1037 cm^−1^ characteristic for the CA were observed (Figure 2a) [27]. The presence of PEG in CA/PEG and CA/PEG/QUE mats resulted in bands at 1100 cm^−1^ characteristic of the PEG ether groups and at 2875 cm^−1^ due to vC–H. In the IR spectrum of CA/PEG/QUE fibers, a shift of the characteristic band for C=O stretching vibrations up to 1747 cm^−1^, compared to the IR spectra of the CA/PEG fibers without QUE (1739 cm^−1^), was detected (Figure 2b). Furthermore, in the IR spectrum of the CA/PEG/QUE mat, there is another shift of the characteristic bands for C=C down to 1600 cm^−1^ and 1508 cm^−1^ compared to the spectrum of the CA/PEG mat (1604 cm^−1^ and 1512 cm^−1^, respectively). A similar shift for the band characteristic of the C=O of the aryl ketone groups of QUE (from 1666 cm^−1^ to 1651 cm^−1^ for the QUE-containing CA/PEG fibers) was also observed. These shifts suggest that hydrogen bonding between CA or PEG and QUE molecules occurs.

### 3.3. Water Contact Angle

**Figure 3:**
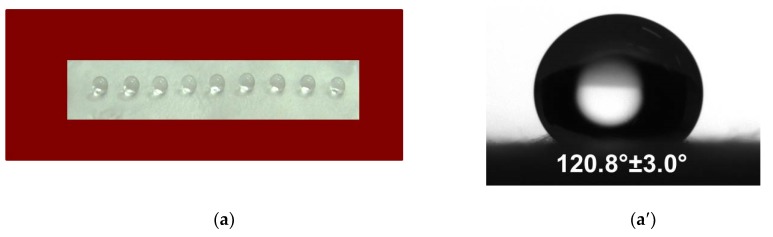

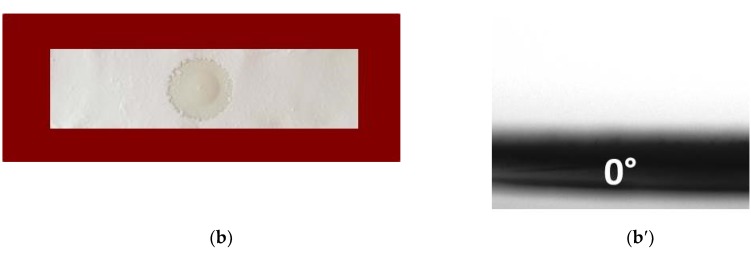
Images of distilled water droplets deposited on the surface of mats: (**a**) and (**a′**). CA; (**b**) and (**b′**). CA/PEG/QUE.

The composition of the fibrous material influences the measured contact angle values. The contact angle value of the CA fibrous mat was 120.08 ± 3.0°. It was found that the CA fibrous material was hydrophobic, and the water droplets remained spherical on it (Figure 3a,a′).

The incorporation of a water-soluble polymer affected the contact angle value. The water contact angle value for the CA/PEG and CA/PEG/QUE mats (Figure 3b,b′) reduced to 0°.

### 3.4. X-ray Diffraction Analysis

**Figure 4:**
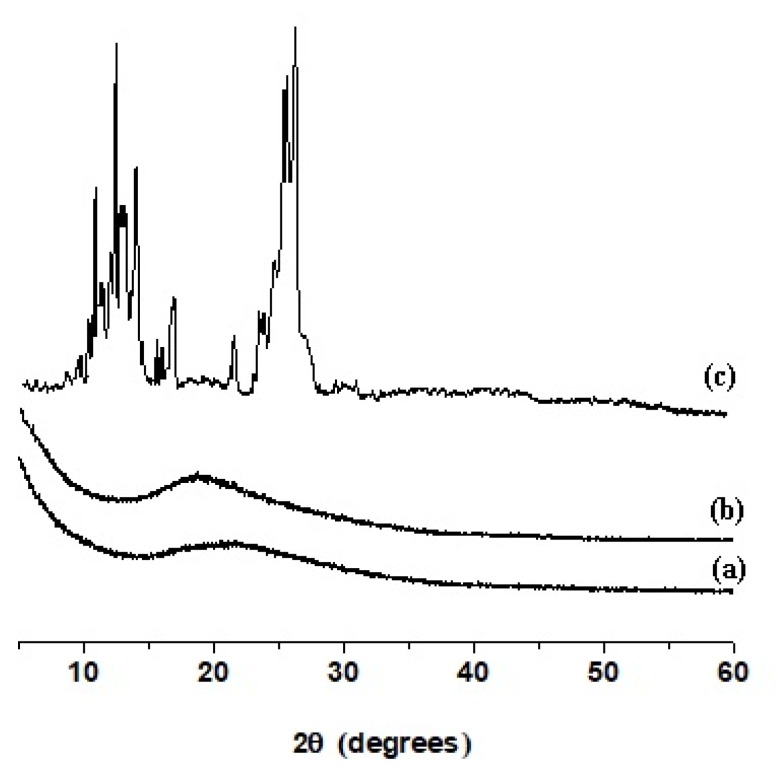
X-ray diffraction pattern of: (a) CA/PEG fibrous material, (b) CA/PEG/QUE fibrous material, and (c) QUE powder.

Figure 4 presents the XRD patterns of the CA/PEG mat (Figure 4a), CA/PEG/QUE mat (Figure 4b) and QUE powder (Figure 4c). X-ray diffraction analysis revealed that the CA/PEG mat was amorphous [28]. Figure 4c shows the X-ray pattern of the QUE (powder), where the main diffraction peaks for quercetin are clearly observed at 2θ = 12.5°, 15.7°, 17.3°, and 27.3°. The presence of these sharp diffraction peaks indicated that the QUE (powder) was highly crystalline. The presence of amorphous halo was recorded in the XRD patterns of CA/PEG/QUE mats Figure 4b. No diffraction peaks for the crystalline phase of QUE were detected, revealing that QUE incorporated in the fibrous material was in amorphous state.

### 3.5. Thermal Characteristics of the Fibrous Mats

**Figure 5:**
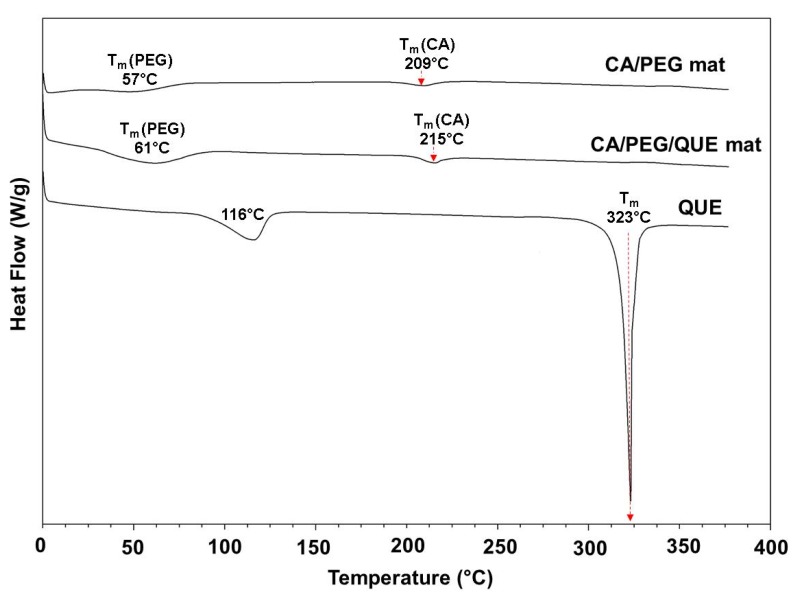
DSC thermograms (first heating run) of: quercetin powder (QUE), CA/PEG/QUE mat, and CA/PEG mat.

The thermal behaviors of the quercetin (powder), CA/PEG, and CA/PEG/QUE fibers were evaluated by DSC analysis. The DSC thermograms (first heating run) of QUE (powder), CA/PEG, and CA/PEG/QUE mats are presented on Figure 5. The melting peak (323 °C) for QUE appeared in the thermogram of the QUE (powder), along with a characteristic broad endothermic peak (116 °C) associated with water molecules loss [29].

The CA/PEG fibers showed a peak for PEG melting point (~ 57 °C). An endothermic peak at 209 °C corresponding to T_m_ of cellulose acetate was also detected. The results are in good agreement with the data presented in the literature concerning the thermal characteristics of cellulose derivative fibers obtained by electrospinning [30,31,32]. The CA/PEG/QUE mat showed a peak for the PEG melting point and a peak for the melting point of CA. However, no peak corresponding to the melting point of QUE was observed in the DSC thermogram of CA/PEG/QUE fibers, which evidenced that the QUE incorporated in the fibers was in amorphous state (Figure 5).

### 3.6. In Vitro Release Profile of Quercetin

In our previous study, we have found that the composition of the polymer matrix played a significant role in the release profile of curcumin from fibrous materials based on cellulose acetate and polyvinyl pyrrolidone [26].

The solubility of quercetin in water is low, lower than 0.01 g/L at 20 °C [33,34]. It has been reported that Tween 80 improves the solubility of poorly soluble compounds (drugs) for better oral administration [35]. Lu et al. have shown that the solubility of quercetin does not change significantly in buffer solutions with different pH compared to its solubility in water [11]. The authors have found that the solubility of quercetin increases by the addition of surfactants, especially by the addition of 0.8% Tween 80.

**Figure 6:**
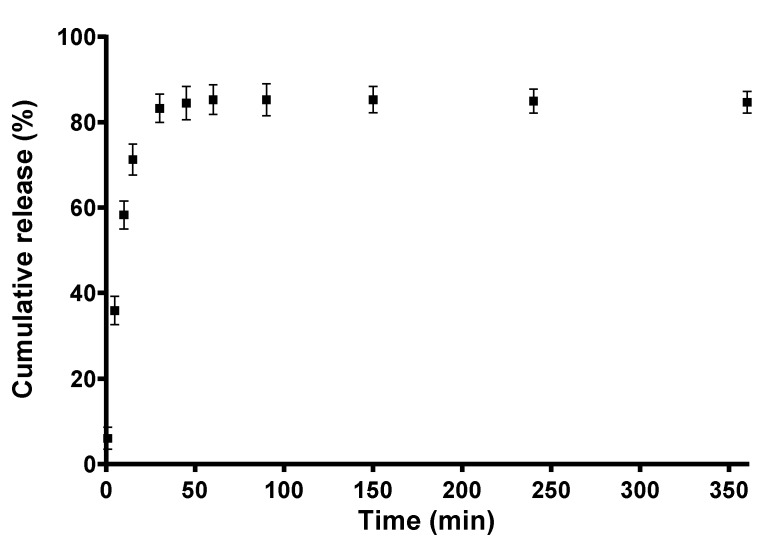
QUE release profile from CA/PEG/QUE fibers. The results are presented as average values from three separate measurements with the respective standard deviation; acetate buffer/Tween 80 (99.2/0.8 *v/v*), pH 5.5, 37 °C, ionic strength 0.1.

In the present study, the release of QUE from the CA/PEG/QUE fibrous mat was performed under model conditions using a procedure for quercetin release in the presence of Tween 80 [11]. The QUE release was carried out in acetate buffer/Tween 80 = 99.2/0.8 *v/v*. Figure 6 shows the release profile of QUE. The amount of QUE released from the CA/PEG/QUE fibers was about ca. 85.3% for 360 min and remained unchanged within 24 h.

The results for the release of quercetin from CA/PEG/QUE fibers were cross-checked by determining the residual amount of QUE after 24 h stay in the release medium. For this purpose, the fibrous mats were dissolved in acetone/water and the absorbance of the obtained solution at 373 nm was recorded. It was found that the total amount of released and residual QUE in the fibrous mats was close to 100%, thus corroborating the findings from the release experiments.

PEG has been reported to improve the solubility of poorly water-soluble biologically active compounds and drugs [35,36]. Several factors affect the release of quercetin from fibrous materials. The simultaneous wetting of the fibrous materials provokes the diffusion of the low-molecular-weight biologically active compound. On the other hand, the wetting depends on the hydrophilic/hydrophobic characteristics and the crystallinity of the polymer/polymers. The release is also affected by the crystalline/amorphous state of the biologically active compound. The diffusion of the biologically active compound also depends on the fiber diameter, the presence of defects and pores. It was found that the hydrophilicity of PEG-containing mats assisted the penetration of the buffer medium as well as the release of biologically active compound quercetin.

### 3.7. Evaluation of the Antioxidant Activity

**Figure 7:**
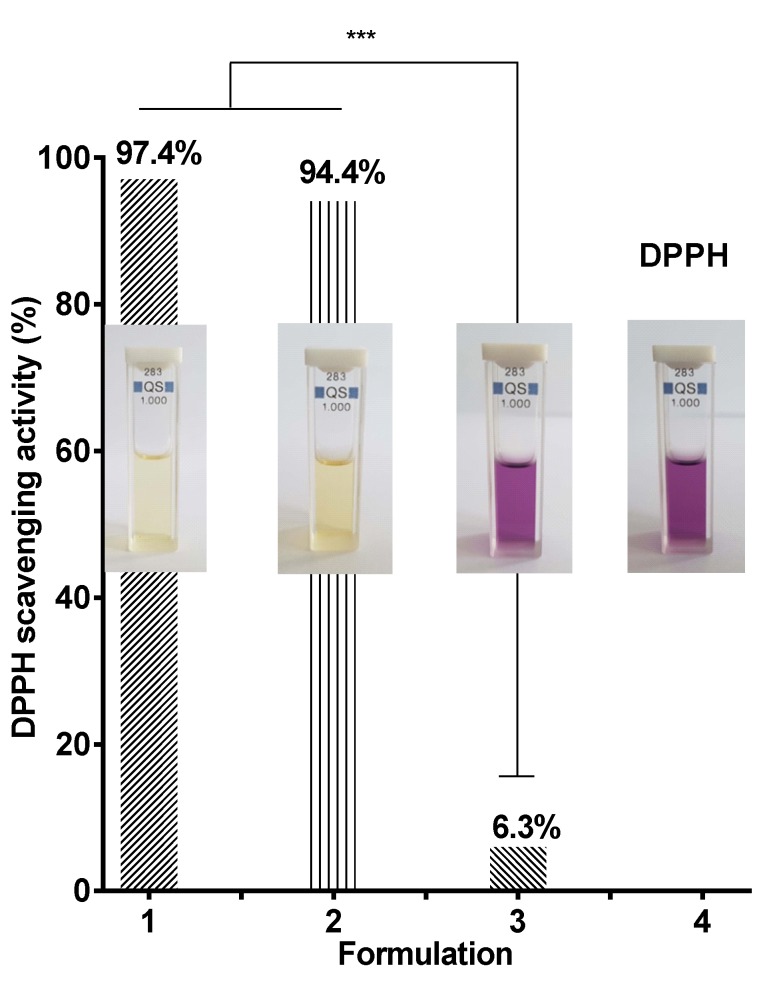
Antioxidant activity of: 1- ethanol solution of quercetin, 2- CA/PEG/QUE mat, and 3 -CA/PEG mat. *** *p* < 0.001. Photos of the corresponding solutions are shown.

It is well known that quercetin exhibits antioxidant and anti-inflammatory activities [37,38]. It can be assumed that its incorporation into CA/PEG fibrous materials will impart them antioxidant activity. Therefore, we evaluated the antioxidant capacity of CA/PEG/QUE mats using 2,2-diphenyl-1-picrylhydrazyl (DPPH•) radical scavenging assay. DPPH• is a stable free radical, and its ethanol solution gives a strong absorption band at 517 nm and a purple color. The reaction was studied using UV-visible spectrophotometry by monitoring the decrease in the absorption of DPPH• in the presence of CA/PEG/QUE mat. For comparison, the antioxidant activity of CA/PEG mats was studied as well. It was found that the CA/PEG mats exhibited very low antioxidant activity (absorbance of DPPH• decreased by approximately 6.3%, Figure 7 (3)). Moreover, the color of the solution of DPPH in contact with CA/PEG mat was not substantially altered, as can be seen from Figure 6. In contrast, after 30 min of contact with DPPH solution, QUE-containing mats exhibited high antioxidant activity (DPPH• absorption decreased by approximately 94.4%). The color of the DPPH solution changed to pale yellow upon contact with CA/PEG/QUE mat (Figure 7 (2)). Moreover, the change in absorption of DPPH solution upon contact with ethanol solution of QUE was similar to that obtained by contact with the fibrous mat containing QUE at the same QUE content (Figure 7 (1)).

### 3.8. MTT Cytotoxicity Assay

In recent years, there has been a rising necessity of developing novel antitumor agents that are more effective and less toxic. Quercetin is such a bioactive compound that is shown to inhibit the growth of various cancer cells [9]. However, cancer treatment could be ineffective in many cases due to relapses and/or the spread of the disease. Thus, the development of new therapeutic strategies remains an important goal in the ongoing battle against this insidious and widespread disease.

**Figure 8:**
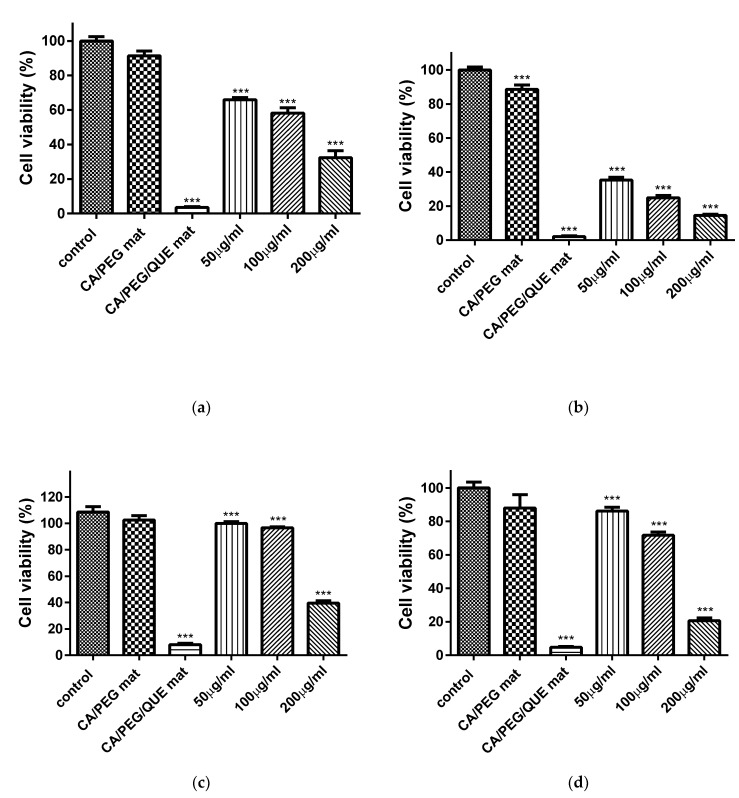
Effect of CA/PEG mat, CA/PEG/QUE mat, and QUE on HeLa tumor cells (**a**,**b**) and SH-4 melanoma cells (**c**,**d**) after 24 h (**a**,**c**) and 48 h (**b**,**d**). Control–untreated HeLa or SH-4 cells; CA/PEG mat; CA/PEG/QUE mat; QUE (50, 100 and 200 µM/L); *** *p* < 0.001.

The cytotoxic effect of QUE on HeLa human cervical cancer cells and SH-4 (human melanoma) was determined by the MTT assay. Figure 8 shows the effect of the materials on the proliferation of HeLa tumor cells and SH-4 skin melanoma cells after 24 h and 48 h. It can be seen that QUE inhibited the cell proliferation. This action was more obvious at higher QUE concentration. The CA/PEG mat did not display any statistically significant antiproliferative activity, as the cell viability was 91.4 ± 3.2% and 99.0 ± 0.3% for HeLa and SH-4 cells, respectively. The percentage of viability of HeLa and SH-4 cells treated with free QUE (200, 100 and 50 µM/L) after 24 h was lower as compared to the non-treated control and CA/PEG fibrous materials. The viability of HeLa cells was 65.9 ± 1.3%, 58.1% ± 2.9%, and 32.2 ± 3.5% for QUE with concentration 50, 100, and 200 µM/L, respectively (Figure 8a). The SH-4 cells viability was 99.7 ± 0.7%, 95.6% ± 0.5% and 39.3% ± 1.5% for QUE with concentration 50, 100, and 50 µM/L, respectively (Figure 8c). For the CA/PEG/QUE mat, after 24 h, the percentage of HeLa and SH-4 cells viability was significantly reduced to 3.5 ± 0.5% and 7.9 ± 0.6% (Figure 8). It is noteworthy that mats containing QUE exhibited much higher antiproliferative activity as compared to CA/PEG mat and free QUE. This is due to the fact that the obtained novel CA/PEG/QUE mats contain the biological active compound in sufficient quantity in order to manifest its antitumor activity and moreover the incorporated PEG facilitates the solubility and the release of QUE. After 48 h (Figure 8b,d of incubation, the inhibition of HeLa and SH-4 cells growth in the presence of CA/PEG/QUE fibrous mat was even higher. Antioxidant activity and kinase inhibition have been proposed as molecular mechanisms of QUE for its anticancer effect [39]. It was observed in the present study that QUE exhibited some higher antiproliferative activity against HeLa cells than that against SH-4 cells. This can be explained with the different susceptibility of the cells to QUE.

### 3.9. Double Staining Assay with AO–EtBr

**Figure 9:**
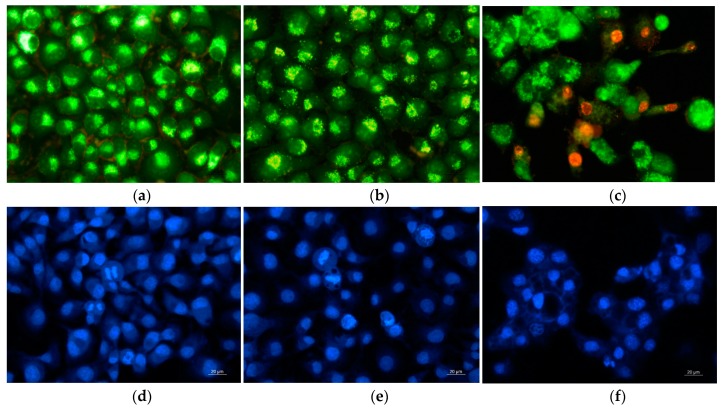
Fluorescence micrographs of AO and EtBr double-stained (**a**–**c**) and stained with DAPI (**d**–**f**) HeLa cancer cells incubated for 24 h (**a**,**d**) untreated cells; after incubation with: (**b**,**e**) CA/PEG mat, (**c**,**f**) CA/PEG/QUE mat; bar = 20 μm.

**Figure 10:**
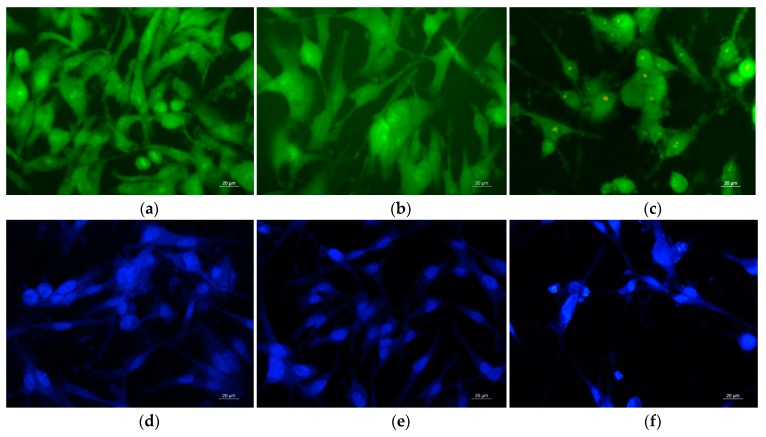
Fluorescence micrographs of AO and EtBr double-stained (**a**–**c**) and stained with DAPI (**d**–**f**) SH-4 skin melanoma cells incubated for 24 h (**a**,**d**) untreated cells; after incubation with: (**b**,**e**) CA/PEG mat, (**c**,**f**) CA/PEG/QUE mat; bar = 20 μm.

The type of cell death was determined by staining of HeLa and SH-4 cells cultivated in the presence of fibrous CA/PEG and CA/PEG/QUE mats with AO and EtBr (1:1 *w/w*) mixture. The stained cells were observed using a fluorescence microscope (Figure 9a–c and Figure 10a–c).

It is well known that the AO passes through intact membranes and emits green fluorescence when interacting with DNA, whereas EtBr passes through the damaged membranes, binds to DNA, and this results in orange staining. The fluorescence micrographs of the observed morphological changes in HeLa and SH-4 cancer cells that resulted from the contact with the fibrous mats are presented in Figure 9a–c and Figure 10a–c, respectively. The controls of untreated cancer cells had predominantly light yellow-green nuclei staining, light green granular cytoplasm (Figure 9a and Figure 10a). After cultivation in the presence of fibrous CA/PEG mat, no significant changes in the staining of nuclei and cytoplasm were observed compared to control untreated cells. However, after contact with the fibrous CA/PEG/QUE mat, both cells lines showed distinct alterations in the cellular and nuclear morphology (Figure 9c and Figure 10c). The nuclei of HeLa cells cultured in presence of CA/PEG/QUE mat revealed chromatin condensation and intensive green or orange staining as well as increased vacuolization of the cytoplasm (Figure 9c). Early apoptotic chromatin condensation changes and membrane blabbing were found in CA/PEG/QUE mat-treated SH-4 cells (Figure 10c). The performed studies have shown that upon contact of cells with QUE-containing mats, the presence of cells with morphological lesions characteristic of early and late stages of apoptosis is observed.

In addition, DAPI staining was applied to examine the changes in the nuclei of HeLa and SH-4 cells in vitro. Fluorescence micrographs of untreated HeLa cells (Figure 9d) show that they possessed intact nuclei, which was oval in shape with diverse size. The nuclei had smooth edges and evenly distributed chromatin. Intact nuclei and evenly distributed chromatin were also observed in the control SH-4 cells. In both cell lines (controls), cell nuclei were observed at different stages of mitosis. Cancer cells that had been in contact with the CA/PEG mat were characterized by morphology of the nuclei, which did not differ from that of the control cancer cells (Figure 9e) and (Figure 10e). Fluorescence micrographs of HeLa and SH-4 cells that were in contact with a QUE-containing mat showed a decrease in cell number and changes in cell morphology of both cell types. Condensation of chromatin (not evenly distributed), fragmentation of the nucleus, and formation of apoptotic bodies (Figure 9f and Figure 10f) revealed the presence of morphological changes. This indicated that quercetin-containing fibrous mats caused cell death via apoptosis. The results show that new CA/PEG/QUE fibrous materials have the potential to be used for local delivery and release of QUE directly into the tumor site, as well as to be used directly on cancerous lesions on the skin.

## 4. Conclusions

Novel fibrous materials with antioxidant and antitumor activities based on cellulose acetate, polyethylene glycol, and quercetin were successfully obtained by electrospinning. It was found that the incorporation of PEG in the polymer matrix resulted in hydrophilization of the material and promoted the QUE release. In addition, it was shown that the quercetin-containing fibrous materials exhibited high antioxidant activity. The in vitro studies performed with cancer cell lines (HeLa and SH-4) showed that quercetin-containing materials exhibited promising antitumor activity. The obtained results revealed that the obtained novel fibrous materials are promising candidates for application in medicine.
